# Editorial: Women in pediatric immunology: 2021

**DOI:** 10.3389/fped.2022.1013758

**Published:** 2022-09-06

**Authors:** Elizabeth Secord

**Affiliations:** Department of Pediatrics, Wayne State University School of Medicine, Detroit, MI, United States

**Keywords:** women, immunology, underrepresented, pediatric, program

An underrepresented group is defined as a subset of a population that holds a smaller percentage within a significant subgroup than the subset holds in the general population. Although women account for approximately half the earth's population, they remain underrepresented in many areas, including most areas of science and medicine. The trend, however, is encouraging. Women have increased from 8% of total science, technology, engineering, and mathematics workers in 1970 to 27% in 2019. Thirty percent of the world's current researchers are women according to data from Forbes. The gap is also narrowing in medicine according to the Association of American Medical Colleges data, which indicates that women comprise 36% of working physicians as of 2019, up from 28% in 2007. As of 2019, medical school classes in the United States had a slightly higher percentage of women than men (50.5%).

The data for immunology are also encouraging. According to US data, women currently comprise 53% of working immunologists. Salaries and promotions have yet to reach the same encouraging data point, however. Women in immunology continue to receive a lower average salary. Advancement in academic settings remains slower for women and they are underrepresented at associate and professorship levels. This is why we decided to publish a special volume of Frontiers in Immunology to spotlight women in Pediatric Immunology.

This volume highlights women in immunology with a variety of research efforts including clinical reports, basic science work, population studies, and a program design, as well as an implementation report for the improvement of inclusivity.

Mitchell et al. present data from Australia and Sweden on the possible prognostic value of plasma signal factors in Langerhans Cell Histiocytosis. Data on differences in biomarkers for immunity and allergic disease development in farming communities vs. non-farming communities in the finger lake region of New York, USA is shared by Järvinen et al. A literature review and reclassification of inborn error of immunity are presented by Carneiro-Sampaio et al. from Sao Paulo, Brazil in collaboration with colleagues at the National Institute of Health, United States. A retrospective analysis of clinical manifestations associated with immune dysregulation in common variable immune deficiency reported in a cohort of children at the University of Medical Sciences in Poznan, Poland is presented by Szczawinska-Poplonyk et al. and reveals a much higher than expected percentage of immune dysregulation.

The final manuscript addresses, at least in part, our reason for this volume and suggests a mechanism for not only equalizing the representation of women but for other underrepresented groups as well. Dimitriades et al. have published a manuscript on the conceptualization and implementation of a program to support women in immunology. A supportive mentorship and sponsorship program supported by women in the Clinical Immunology Society is delineated, beginning with a survey, followed by education of the involved population.

The breadth of work in this volume is inspirational in its content and its implication that with time, persistence, and heightened awareness, this strategy of support in pediatric immunology research can be continued for women and other underrepresented groups.

## Author contributions

The author confirms being the sole contributor of this work and has approved it for publication.

## Conflict of interest

The author declares that the research was conducted in the absence of any commercial or financial relationships that could be construed as a potential conflict of interest.

## Publisher's note

All claims expressed in this article are solely those of the authors and do not necessarily represent those of their affiliated organizations, or those of the publisher, the editors and the reviewers. Any product that may be evaluated in this article, or claim that may be made by its manufacturer, is not guaranteed or endorsed by the publisher.

